# Identification of a novel YAP–14-3-3ζ negative feedback loop in gastric cancer

**DOI:** 10.18632/oncotarget.18011

**Published:** 2017-05-19

**Authors:** Bin Zhang, Aihua Gong, Hui Shi, Qingli Bie, Zhaofeng Liang, Peipei Wu, Fei Mao, Hui Qian, Wenrong Xu

**Affiliations:** ^1^ Key Laboratory of Laboratory Medicine of Jiangsu Province, School of Medicine, Jiangsu University, Zhenjiang, Jiangsu, P.R. China; ^2^ The Affiliated Hospital, Jiangsu University, Zhenjiang, Jiangsu, P.R. China

**Keywords:** YAP, 14-3-3ζ, MDM2, hippo, gastric cancer

## Abstract

Growing evidence indicates that 14-3-3ζ and yes-associated protein (YAP) substantially promote tumorigenesis and tumor development. However, the regulatory mechanism underlying these two proteins remains unknown. Herein, we report a new regulatory role of 14-3-3ζ in the phosphorylation of YAP and the feedback inhibition of 14-3-3ζ by YAP. YAP and 14-3-3ζ expression exhibited a negative correlation in gastric cancer (GC) tissues. Moreover, patients with higher YAP and lower 14-3-3ζ expression had poor prognoses. Studies have revealed that 14-3-3ζ promotes cytoplasmic retention and suppresses the transcriptional activity of YAP by inducing its phosphorylation. Furthermore, we observed that the overexpression of YAP significantly reduced the expression of 14-3-3ζ by inducing its ubiquitination. YAP, 14-3-3ζ, and mouse double minute 2 homolog (MDM2) were colocalized, and the knockdown of MDM2 by siRNA attenuated the YAP-induced decrease of 14-3-3ζ. The binding of 14-3-3ζ and MDM2 was also restrained when the expression of YAP was interfered. Our results indicated the presence of a 14-3-3ζ–YAP negative regulatory feedback loop, which has a crucial role in cell proliferation and survival and is a potential target for the clinical treatment of GC.

## INTRODUCTION

The incidence of gastric cancer (GC) has decreased over the past few decades; however, it remains the fourth most common cancer and second leading cause of death from cancer worldwide [1–3]. Over the past decades, considerable effort has been devoted to elucidating its underlying mechanisms and discovering novel diagnostic biomarkers and therapeutic targets. However, treatments for GC continue to be ineffective [4], and its mortality rate remains high [5]. Although extensive knowledge has been obtained regarding biomarkers and the therapeutic targets that affect cancer cell properties, few studies have investigated the role of mutual regulation among these proteins in GC or their underlying mechanisms.

The Hippo pathway plays a critical role in organ size control by regulating cell growth, proliferation, and apoptosis [6–9]. Yes-associated protein 1 (YAP) and transcriptional coactivator with PDZ-binding motif (TAZ) function as the key downstream effectors of the Hippo pathway [10]. In mammals, a key serine (S127) of YAP is phosphorylated by large tumor suppressor (LATS), which confines YAP within the cytoplasm where it can no longer target gene expression [11, 12]. The abnormal activation of YAP, an oncoprotein, has been observed frequently in various cancer types [13–16]. YAP is also strongly expressed in gastric adenocarcinomas, and the knockdown of YAP may inhibit GC cell proliferation [17]. These findings reveal that the inhibitory molecules of YAP may be effective therapeutic targets in GC.

The 14-3-3 protein family is expressed in various organs and is highly conserved in all eukaryotic organisms; it has seven different 14-3-3 isoforms in humans [18, 19]. All 14-3-3 proteins can activate or inhibit the activity of target proteins by changing the protein conformation, increasing or decreasing protein stability, facilitating protein complex formation, or altering protein subcellular localization [20, 21]. In recent years, the role of 14-3-3 proteins in malignant tumors has been increasingly reported. For example, 14-3-3ζ was observed to be upregulated in prostate cancer and facilitate the progression of prostate cancer [22]. Furthermore, 14-3-3ζ overexpression was found to be involved in PI3K triggered AKT phosphorylation and the cancer cell invasion in human breast cancer caused by ionizing radiation [23, 24].

A recent study reported that 14-3-3ζ can accelerate the progression of breast cancer by changing the function of TGF-β [25]. The aforementioned studies have confirmed the facilitating role of 14-3-3ζ in prostate and breast cancers. Moreover, recent studies reporting the function of 14-3-3 proteins in the Hippo pathway have revealed that they bind to p127-YAP and induce YAP cytoplasmic retention [26, 27]. A recent study reported that 14-3-3σ can inhibit keratinocyte proliferation and promote differentiation by inducing YAP cellular localization [28]. Our results also confirmed that exosomal 14-3-3ζ restricted cell expansion due to YAP [29]. These studies have all indicated that 14-3-3ζ may perform another role by modulating YAP in GC.

In this study, we observed a strong correlation between 14-3-3ζ and YAP expression and a contrasting pattern in gastric tissues. According to the results of a GC tissue microarray, GC patients had poor prognoses if they displayed higher levels of YAP protein expression than of 14-3-3ζ expression. This result indicated a potential regulatory mechanism between 14-3-3ζ and YAP. The overexpression of YAP in gastric cells enhanced its proliferation; however, the same effect was not observed for 14-3-3ζ. Additional studies have revealed that 14-3-3ζ induced YAP phosphorylation at the serine 127 (Ser127) site. YAP also induced the ubiquitination and degradation of 14-3-3ζ by mediating the binding of mouse double minute 2 homolog (MDM2; an E3 ubiquitin ligase) and 14-3-3ζ. Thus, a remarkable negative feedback loop between YAP and 14-3-3ζ was identified in GC.

## RESULTS

### YAP and 14-3-3ζ exhibited contrasting expression patterns in GC tissues that correlated with patients’ prognoses

**Figure 1:**
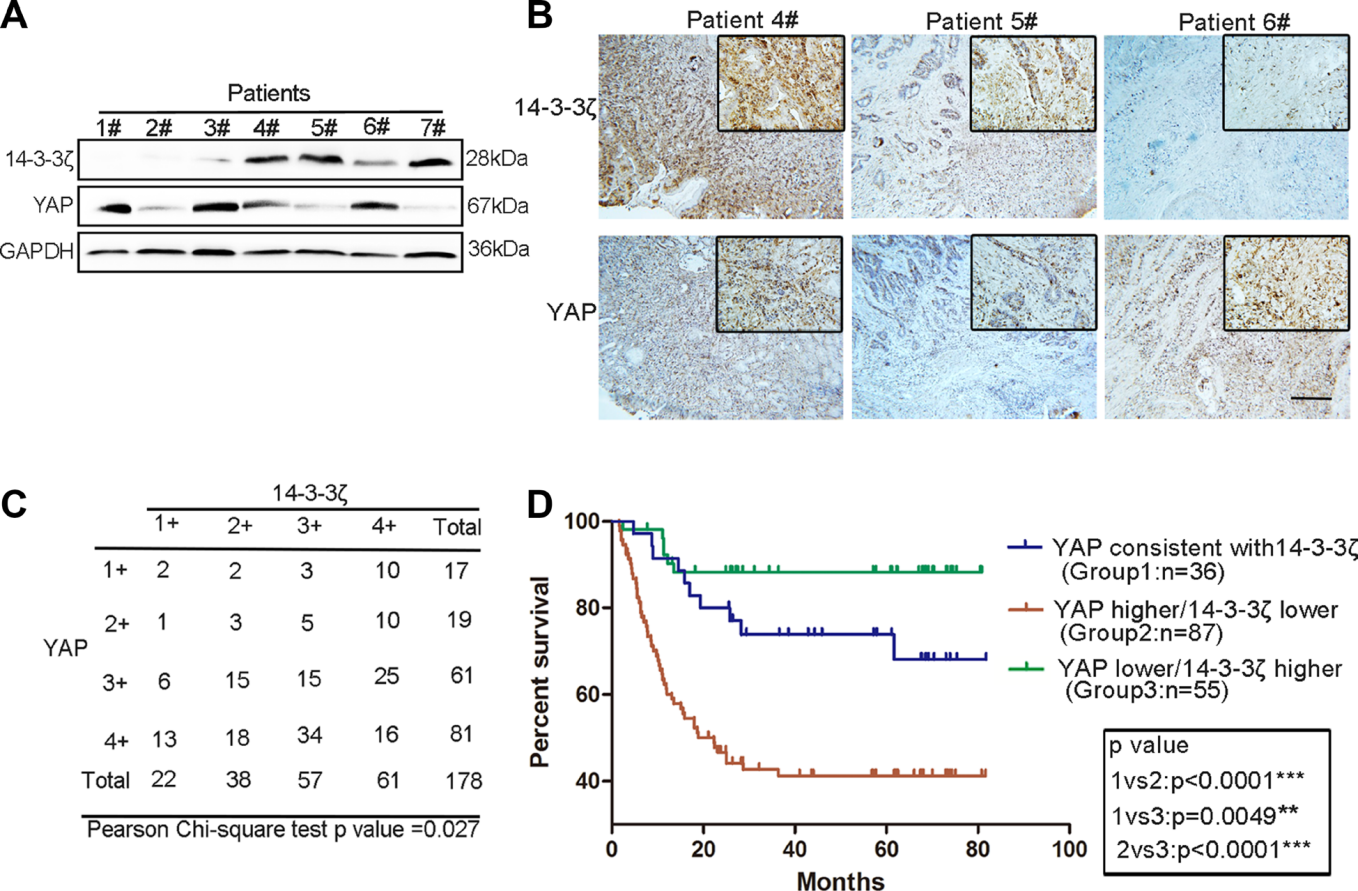
Contrasting expression patterns of YAP and 14-3-3ζ in GC tissues were associated with patient prognoses (**A**) Western blotting assay of YAP and 14-3-3ζ protein levels in GC. (**B**) Representative images of YAP and 14-3-3ζ histochemical staining in surgical specimens from GC tissues. The patient numbers used in the immunohistochemistry analysis correspond to those used in the western blot in Figure 1A. Scale bar = 100 μm. (**C**) Statistical analysis of the correlation between YAP and 14-3-3ζ expression in GC tissue microarray using the chi-square test. The scores of YAP and 14-3-3ζ expression (1+, 2+ 3+ and 4+) are shown in Supplementary Figure 1A. (**D**) Kaplan–Meier analysis indicates a correlation between the cumulative survival and different expression patterns of YAP and 14-3-3ζ among patients according to the results of GC tissue microarray. Statistical significance was assessed using the log-rank test. As in Figure 1C, when the scores of YAP and 14-3-3ζ expression were equal in GC tissue, YAP was defined as consistent with 14-3-3ζ. When the score of YAP expression was higher than that of 14-3-3ζ, it was defined as higher than 14-3-3ζ. When YAP expression score was lower than that of 14-3-3ζ, it was defined as lower than 14-3-3ζ.

Our early results revealed that 14-3-3ζ promoted YAP phosphorylation and thus restricted cell proliferation [29]. This result strongly suggested that 14-3-3ζ is not only a companion molecule but also a regulator of the Hippo pathway. Considering the critical role of YAP in cancer, we hypothesized that a novel regulatory mechanism exists between 14-3-3ζ and YAP in GC. To validate our hypothesis, we detected 14-3-3ζ and YAP in a sample of GC tissues through western blot analysis. YAP and 14-3-3ζ exhibited contrasting expression patterns in the GC tissues (Figure 1A and Supplementary Figure 1A). We reexamined the expression of 14-3-3ζ and YAP and confirmed the expression pattern through immunohistochemistry (Figure 1B). Furthermore, to confirm this finding and account for the heterogeneity within GC tissues, we detected 14-3-3ζ and YAP expression in a GC tissue microarray. The grading of YAP and 14-3-3ζ expression is shown in Supplementary Figure 1C, with low expression (grades 1 and 2), and high expression (grades 3 and 4). Statistical analysis of the tissue microarray revealed a significant correlation between YAP and 14-3-3ζ expression (Figure 1C). As shown in the Kaplan–Meier survival graph (Figure 1D), regardless of the inherent gene expression level, GC patients with higher grades of YAP expression than of 14-3-3ζ expression had poor prognoses, with a survival rate of approximately 40%. By contrast, the survival rate of patients with GC who had low grades of YAP expression and high grades of 14-3-3ζ expression was the highest (approximately 90%; Figure 1D). Tables 1 and 2 show that the expression of YAP and 14-3-3ζ were associated with tumor size (*p* = 0.021 and 0.044, respectively). However, no significant differences were observed between YAP, 14-3-3ζ.level and histological grade (*p* = 0.412 and 0.383, respectively) and T categories (*p* = 0.425 and 0.590, respectively), gender (*p* = 0.255 and 0.949, respectively), age (*p* = 0.205 and 0.923), lymph node metastasis (N factor; *p* = 0. 0.097 and 0.458, respectively), or tumor node metastasis stages (*p* = 0.886 and 0.391, respectively). These results indicate a contrasting expression pattern of YAP and 14-3-3ζ and reveal a linear correlation in GC tissues that may affect the prognoses of patients with GC.

**Table 1:** Correlation between clinicopathological factors and YAP IHC staining in GC samples

Factor	Number (%)	YAP IHC staining		*P*-value
		Low	High	
		group	group	
Age (years)				
> 60	81 (45.5%)	13	68	0.205
≤ 60	97 (54.5%)	23	74	
Gender				
Male	130 (73%)	29	101	0.255
Female	48 (27%)	7	41	
Size (cm)				
> 5	85 (47.8%)	11	74	0.021*
≤ 5	93 (52.2%)	25	68	
Histological grade				
Well + moderately	75 (42.1%)	13	62	0.412
Poorly + signet	103 (57.9%)	23	80	
T grade				
T1 + T2	49 (27.5%)	8	41	0.425
T3 + T4	129 (72.5%)	28	101	
Lymph node metastasis (N factor)				
Absent (N0)	63 (35.4%)	17	46	0.097
Present (N1–N3)	115 (64.6%)	19	96	
Stage				
I/II	97 (54.5%)	20	77	0.886
III/IV	81 (45.5%)	16	65	

*P < 0.05.

**Table 2:** Correlation between clinicopathological factors and 14-3-3ζ IHC staining in GC samples

Factor	Number (%)	14-3-3ζ IHC staining		*P*-value
		Low	High	
		group	group	
Age (years)				
> 60	81 (45.5%)	27	54	0.923
≤ 60	97 (54.5%)	33	64	
Gender				
Male	130 (73%)	44	86	0.949
Female	48 (27%)	16	32	
Size (cm)				
> 5	85 (47.8%)	35	50	0.044*
≤ 5	93 (52.2%)	25	68	
Histological grade				
Well + moderately	75 (42.1%)	28	47	0.383
Poorly + signet	103 (57.9%)	32	71	
T grade				
T1 + T2	49 (27.5%)	15	34	0.590
T3 + T4	129 (72.5%)	45	84	
Lymph node metastasis(N factor)				
Absent (N0)	63 (35.4%)	19	44	0.458
Present (N1–N3)	115 (64.6%)	41	74	
Stage				
I/II	97 (54.5%)	30	67	0.391
III/IV	81 (45.5%)	30	51	

*P < 0.05.

### YAP and 14-3-3ζ expression had contrasting effects on GC cell proliferation

**Figure 2:**
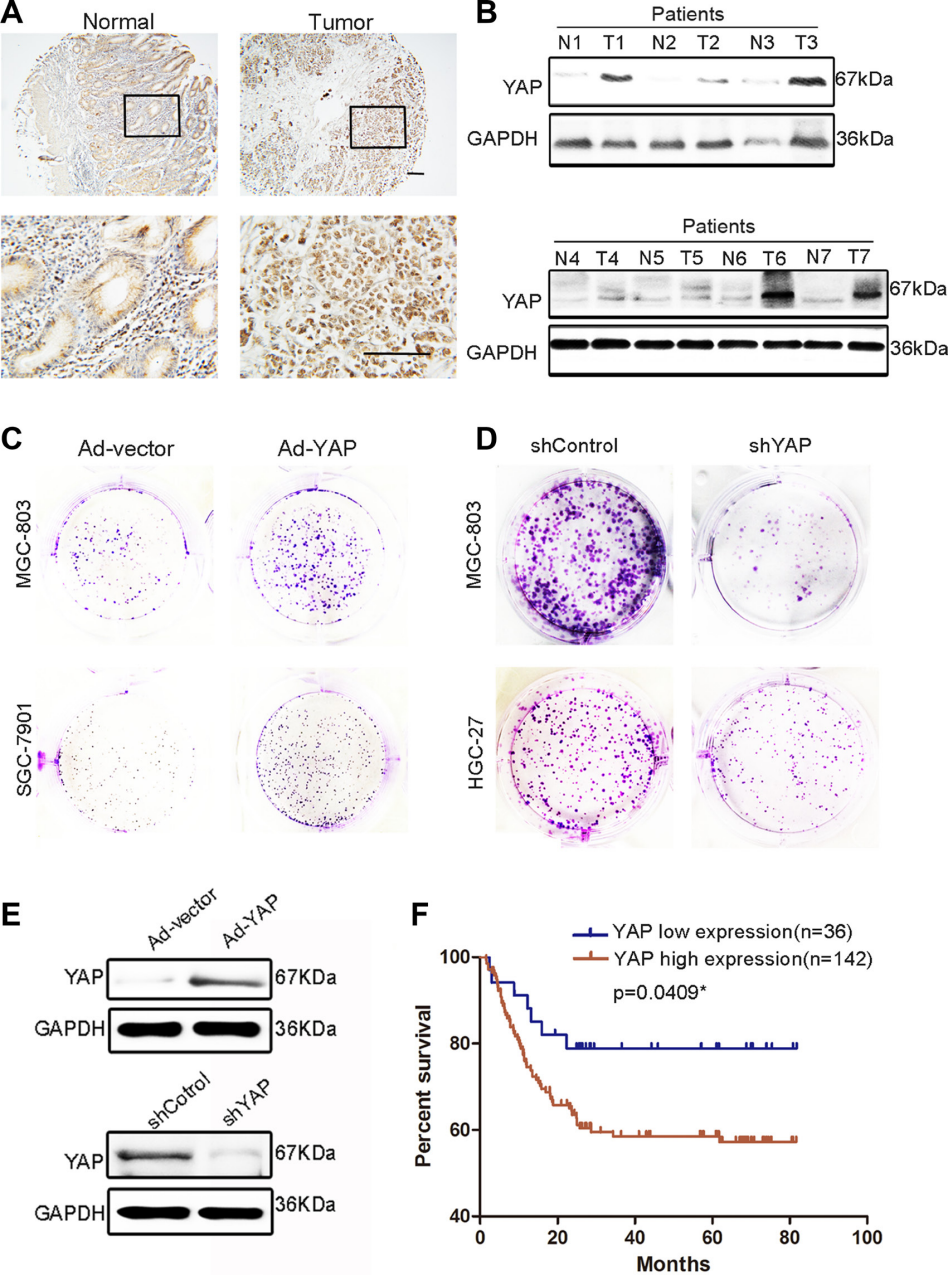
YAP expression in GC tissues and paired adjacent normal tissues and its proliferation-promoting role in GC cells (**A**) Representative images of YAP histochemical staining in surgical specimens from normal and GC tissues. Scale bar = 100 μm. (**B**) Western blotting assay of YAP protein levels in GC (T) and corresponding adjacent tissues (N). (**C**) Representative images of colony formation in blank vector (Ad-vector)- and YAP-overexpressing adenovirus (Ad-YAP)-transfected MGC-803 and SGC-7901 cells. The Ad-YAP and Ad-vector transfected cells were cultured for 5 days. (**D**) Representative images of colony formation in shYAP and shControl-transfected MGC-803 and HGC-27 cells. shYAP and shControl-transfected cells were cultured for 10 days. (**E**) Validation of the knockdown or overexpression effect in Figure 2C and 2D through western blotting. (**F**) The grading of YAP and 14-3-3ζ expression is shown in Supplementary Figure 1B, with low expression (grades 1 and 2) and high expression (grades 3 and 4). Kaplan–Meier analysis shows a correlation between cumulative survival and YAP expression levels in patients with GC. Statistical significance was assessed using the log-rank test.

Although several studies have reported that YAP plays a crucial role in GC and other malignant tumors [17, 26, 27], we attempted to confirm the action of YAP in GC in our system. Western blotting and immunohistochemistry indicated that YAP expression was significantly higher in GC tissues compared with that in adjacent tissues (Figure 2A and 2B). YAP overexpression strongly promoted MGC-803 and SGC-7901 cell colony-forming ability, which was replicated in MKN-45 and HGC-27 cells (Figure 2C and Supplementary Figure 2A). The knockdown of YAP suppressed the colony-forming ability of HGC-27 and MGC-803 cells (Figure 2D). However, the overexpression of YAP did not promote the migration ability of MGC-803 cells (Supplementary Figure 2C). Before performing these experiments, we validated the efficiency of YAP overexpression and knockdown (Figure 2E). These results indicated that YAP promoted GC cell proliferation and that high YAP expression in GC tissues is correlated with poor prognosis (Figure 2F).

**Figure 3:**
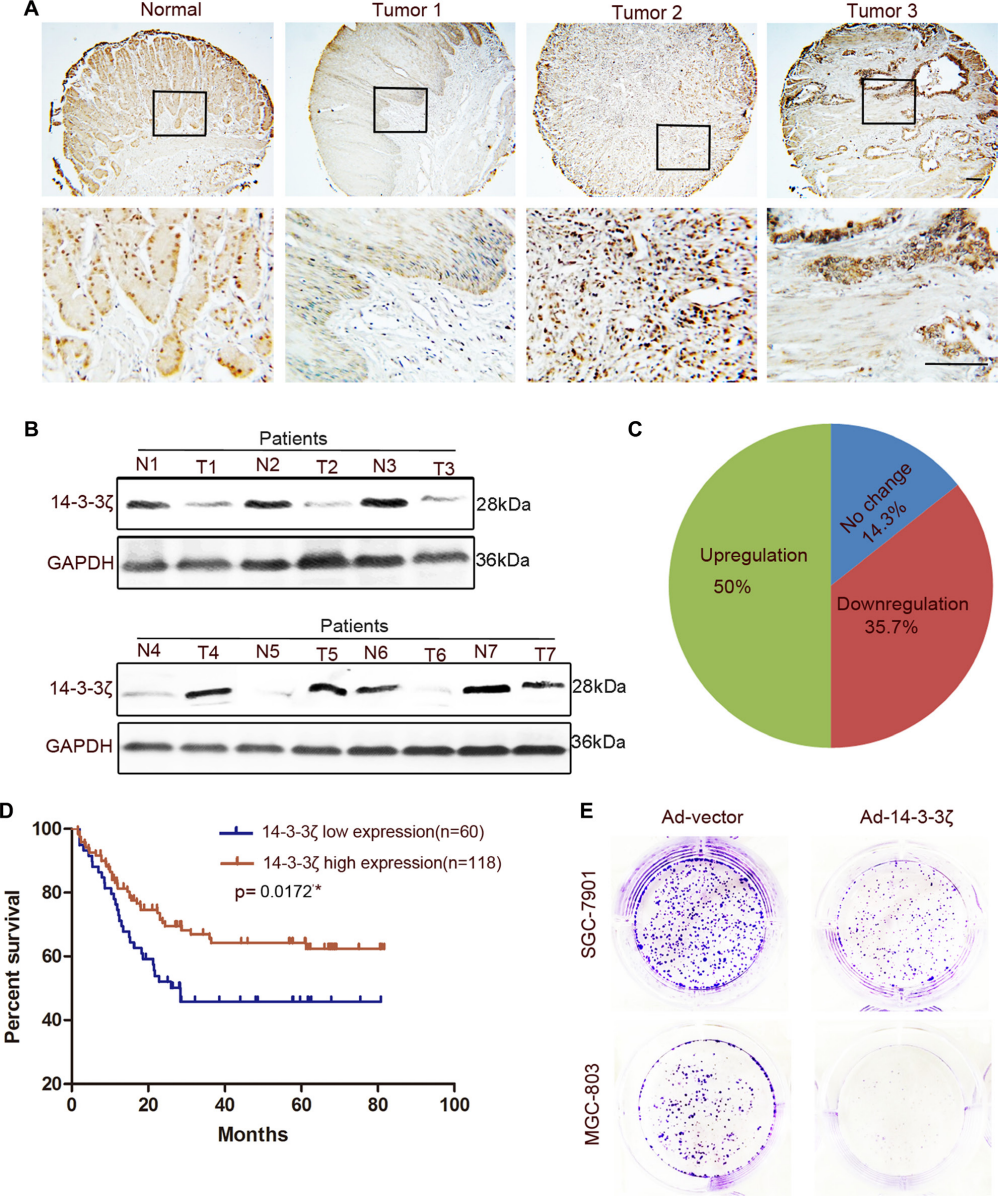
Expression of 14-3-3ζ was detected in GC tissues and paired adjacent normal tissues and its role in GC cells was revealed (**A**) Representative images of 14-3-3ζ histochemical staining in surgical specimens from normal and GC tumor tissues. Scale bar = 100 μm. (**B**) Western blotting assay of 14-3-3ζ protein levels in GC (T) and corresponding adjacent tissues (N). (**C**) The expression of 14-3-3ζ in GC tissue microarray compared with that in normal tissues. (**D**) The grading of YAP and 14-3-3ζ expression is shown in Supplementary Figure 1B, with low expression (grades 1 and 2), and high expression (grades 3 and 4). Kaplan–Meier analysis indicates a correlation between cumulative survival and 14-3-3ζ expression levels in patients with GC. Statistical significance was assessed using the log-rank test. (**E**) Representative images of colony formation in Ad-vector- or Ad-14-3-3ζ-transfected MGC-803 and SGC-7901 cells. The transfected cells were cultured for 5 days.

Nishimura et al. reported that 14-3-3ζ expression was high in GC tissues and was associated with tumor cell proliferation and the malignant outcomes of gastric carcinoma [30]. However, we observed that not all GC tissues expressed a high level of 14-3-3ζ. The expression of 14-3-3ζ was lower in part of the tumor tissues than in the normal gastric mucosa (Figure 3A and 3B). To exclude the effects on our results of heterogeneity among GCs, we expanded the sample sizes by using a tissue microarray. The tissue microarray results indicated that only 50% of the GC tissue samples (89/178) exhibited higher 14-3-3ζ expressions than those of adjacent tumor tissues (Figure 3C). This result was consistent with that reported by Nishimura et al. However, 14-3-3ζ expression was low in 35.7% of GC tumor tissues (64/178; Figure 3C). More importantly, 14-3-3ζ differed from YAP in that its low expression was correlated with poor prognosis (Figure 3D). In addition, 14-3-3ζ overexpression markedly inhibited MGC-803 and SCG-7901 cell colony-forming ability (Figure 3E). This result was also confirmed in MKN-45 and HGC-27 cells (Supplementary Figure 3A). Similarly, 14-3-3ζ did not inhibit the migration of MGC-803 cells (Supplementary Figure 3B).

### 14-3-3ζ inhibited the activation of YAP by inducing its serine 127 phosphorylation and cytoplasmic retention

**Figure 4:**
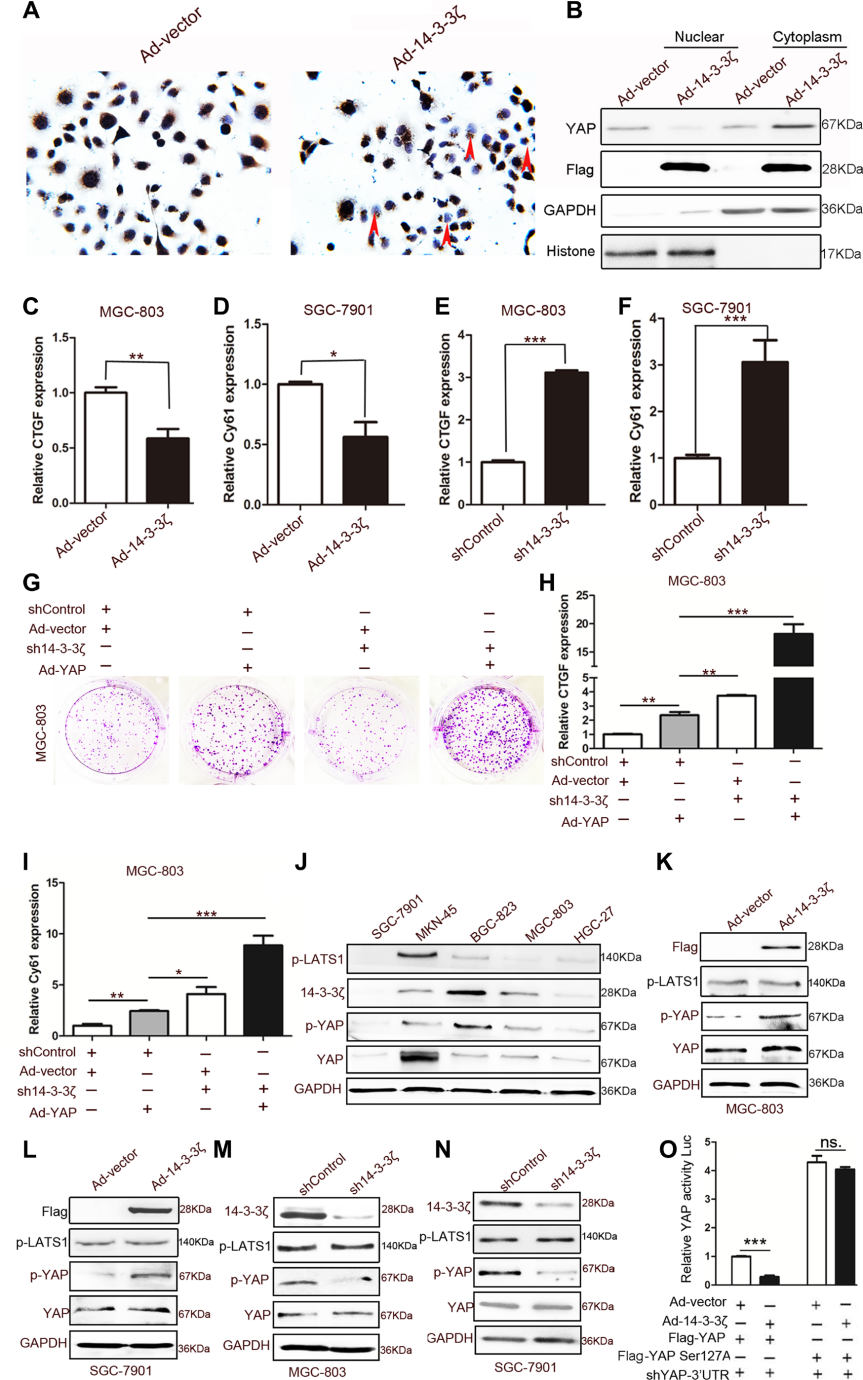
Effect of 14-3-3ζ on YAP (**A**) Representative images of YAP histochemical staining in Ad-vector- and Ad-14-3-3ζ-transfected MGC-803 cells. The red arrows indicate cells without Ad-14-3-3ζ-transfected MGC-803 for which YAP expression is negative. (**B**) Cytoplasmic and nuclear fractions were prepared from MGC-803 cells transfected with blank vector or 14-3-3ζ-overexpressing adenovirus, and YAP protein levels were determined through western blotting. (**C**) and (**D**) Quantitative analyses of CTGF and Cy61 mRNA in MGC-803 cells transfected with vector or 14-3-3ζ-overexpressing adenovirus (*n* = 3; *p < 0.05; **p < 0.01). (**E**) and (**F**) Quantitative analyses of CTGF and Cy61 mRNA in MGC-803 cells transfected with shControl or sh14-3-3ζ lentivirus (*n* = 3; ***p < 0.001). (**G**) Representative images of colony formation in MGC-803 cells transfected with a blank vector or YAP-overexpressing adenovirus and shControl or sh14-3-3ζ lentivirus. The transfected cells were cultured for 5 days. (**H**) and (**I**) Quantitative analyses of CTGF and Cy61 mRNA in MGC-803 cells transfected with blank vector or YAP-overexpressing adenovirus and shControl or sh14-3-3ζ lentivirus (*n* = 3; *p < 0.05; **p < 0.01; ***p < 0.001) (**J**) Western blotting assay of 14-3-3ζ, YAP, p-LTAS1, and GAPDH expression in human GC cell lines. (**K**) Western blotting assay for Flag (the tag of 14-3-3ζ overexpression), p-LATS1, p-YAP, and YAP expression in Ad-vector- or Ad-14-3-3ζ-transfected MGC-803 cells. (**L**) Western blotting assay for Flag (the tag of 14-3-3ζ overexpression), p-LATS1, p-YAP, and YAP expression in Ad-vector or Ad-14-3-3ζ transfected SGC-7901 cells. (**M**) Western blotting assay for 14-3-3ζ, p-LATS1, p-YAP, and YAP expression in 14-3-3ζ-disrupted or control MGC-803 cells for 48 h. (**N**) Western blotting assay for 14-3-3ζ, p-LATS1, p-YAP, and YAP expression in 14-3-3ζ-disrupted or control SGC-7901 cells for 48 h. (**O**) 293T cells were transfected with the indicated adenoviruses and plasmids, and the luciferase activities were measured and normalized to that of Renilla. (shYAP-3′UTR is shRNA that is targeted to endogenous YAP; *n* = 3; ***p < 0.001).

Studies on the role of 14-3-3 proteins in the Hippo pathway have revealed that they bind to p127-YAP and induce the cytoplasmic retention of YAP [9, 10]. However, Sambandam et al. were the first to demonstrate that the change of 14-3-3 expression can increase the cytoplasmic retention of YAP. They found that 14-3-3σ regulates keratinocyte proliferation and differentiation by modulating YAP cellular localization [28]. We speculated that, as a 14-3-3 protein isoform, 14-3-3ζ may induce cytoplasmic retention through some novel mechanisms. As anticipated, 14-3-3ζ overexpression promoted YAP translocation from the nucleus to the cytoplasm in SGC-7901 cells (Figure 4A and 4B), and the knockdown of 14-3-3ζ reversed this phenomenon (Supplementary Figure 4A). Moreover, 14-3-3ζ overexpression suppressed the expression of the targets of YAP, namely CTGF and Cy61 in MGC-803 and SGC-7901 cells, respectively (Figure 4C and 4D), and the knockdown of 14-3-3ζ promoted the expression of these targets (Figure 4E and 4F). The ability of YAP to promote the colony-forming ability of MGC-803 was enhanced by the knockdown of 14-3-3ζ (Figure 4G). In addition, the expansion indicators proliferating cell nuclear antigen (PCNA), cyclin-D1, and cyclin-D3 produced similar outcomes (Figure 4H and Supplementary Figure 4B). The expressions of CTGF and Cy61 were also enhanced by the shRNA lentivirus of 14-3-3ζ (Figure 4H and 4I). These results strongly suggested that 14-3-3ζ negatively regulated the nuclear translocation of YAP and its transcriptional activity.

Recent studies have revealed that the phosphorylation of the YAP-Ser127 site is the main mechanism of the cytoplasmic retention of YAP [9, 10]. To determine the mechanism of action through which 14-3-3ζ inhibits YAP, we detected the expression of YAP, p-YAP, 14-3-3ζ, and the YAP upstream kinase p-LATS1 in five GC cell lines with different degrees of differentiation. The results revealed that the phosphorylation of YAP at the Ser127 site was consistent with the trend of 14-3-3ζ expression rather than that of p-LATS (Figure 4J). Consequently, we hypothesized that 14-3-3ζ induces the phosphorylation of YAP at Ser127 to inhibit its activation. Both the overexpression and knockdown of 14-3-3ζ validated our hypothesis; 14-3-3ζ affected the phosphorylation of YAP in MGC-803 and SGC-7901 cells (Figure 4K–4N). The role of p127-YAP was further explored through site-directed mutagenesis. Notably, the mutation of Ser127 nearly supressed the inhibitory effect of 14-3-3ζ on YAP transcriptional activity (Figure 4O). The phosphorylation of YAP induced by 14-3-3ζ depended on LATS1, because the knockdown of LATS1 fully accounts for the phosphorylation of YAP due to 14-3-3ζ (Supplementary Figure 4C). The knockdown of 14-3-3ζ expression disturbed the interaction between YAP and pLATS1 in MGC-803 cells (Supplementary Figure 4D).

### YAP reduced the expression of 14-3-3ζ

**Figure 5:**
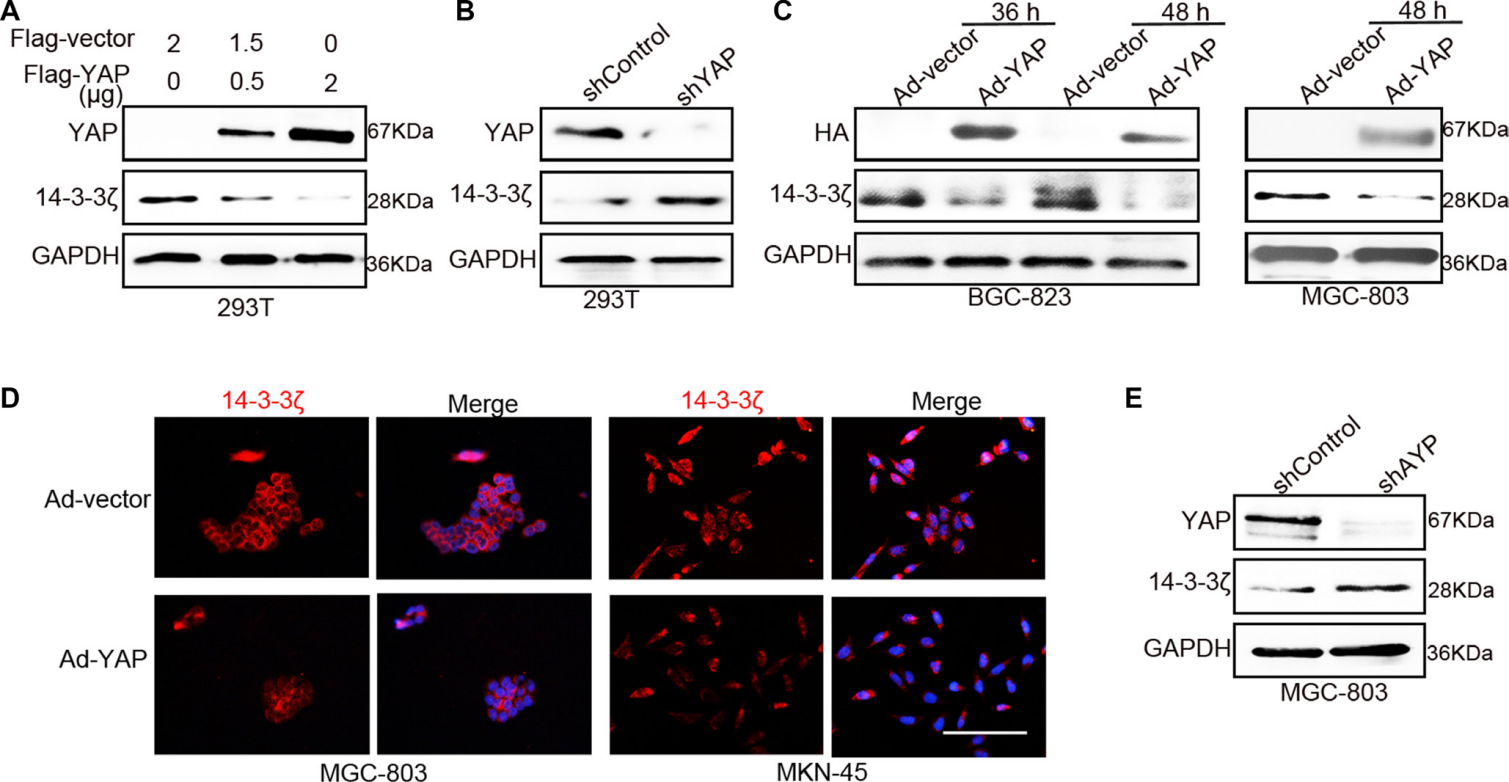
YAP inhibited the expression of 14-3-3ζ (**A**) Western blotting assay for 14-3-3ζ expression after transfecting with Flag-vector or -YAP plasmid in 293T cells for 60 h. (**B**) The expression of 14-3-3ζ was detected in YAP-disrupted or control 293T cells through western blotting after 48 h. (**C**) Western blotting assay for 14-3-3ζ expression in BGC-823 and MGC-803 cells after transfecting with vector or YAP-overexpressing adenovirus at the indicated time point. (**D**) Representative images of 14-3-3ζ immunofluorescence staining confirmed the results in (c). Red indicates 14-3-3ζ; blue indicates the nuclear dye hoechst33342; “merge” indicates that 14-3-3ζ is merged with hoechst33342. Scale bar = 100 μm. (**E**) Western blotting assay for 14-3-3ζ expression after transfecting with shControl or sh14-3-3ζ lentivirus for 48 h in MGC-803 cells.

We found that 14-3-3ζ induced the phosphorylation of YAP but did not inhibit its complete expression. In brief, the contrasting expression patterns of YAP and 14-3-3ζ in GC tissues required further elucidation. Because of these results, we suspected that the effect of YAP on 14-3-3ζ expression involves a negative feedback loop. Therefore, we overexpressed YAP in 293T cells and found that YAP markedly reduced the expression of 14-3-3ζ in a concentration-dependent manner (Figure 5A). Although 14-3-3σ can regulate YAP in other cancer cells [31], the overexpression of YAP did not decrease other 14-3-3 proteins such as 14-3-3η, 14-3-3ε, 14-3-3γ, and 14-3-3σ (Supplementary Figure 5A). Hence, the expressions of other 14-3-3 proteins including 14-3-3σ were not detected in GC tissue. The knockdown of YAP by shRNA promoted the expression of 14-3-3ζ in 293T cells (Figure 5B). This result was confirmed in BGC-823, MGC-803, and MKN-45 cells (Figure 5C and Supplementary Figure 5B). Moreover, immunofluorescence results revealed that YAP overexpression reduced 14-3-3ζ expression in MGC-803, MKN-45, and BGC-823 cells (Figure 5D and Supplementary Figure 5C). Similarly, YAP downregulation promoted 14-3-3ζ expression in MGC-803 cells (Figure 5E).

### YAP recruited MDM2 to 14-3-3ζ and induced the ubiquitination of 14-3-3ζ

**Figure 6:**
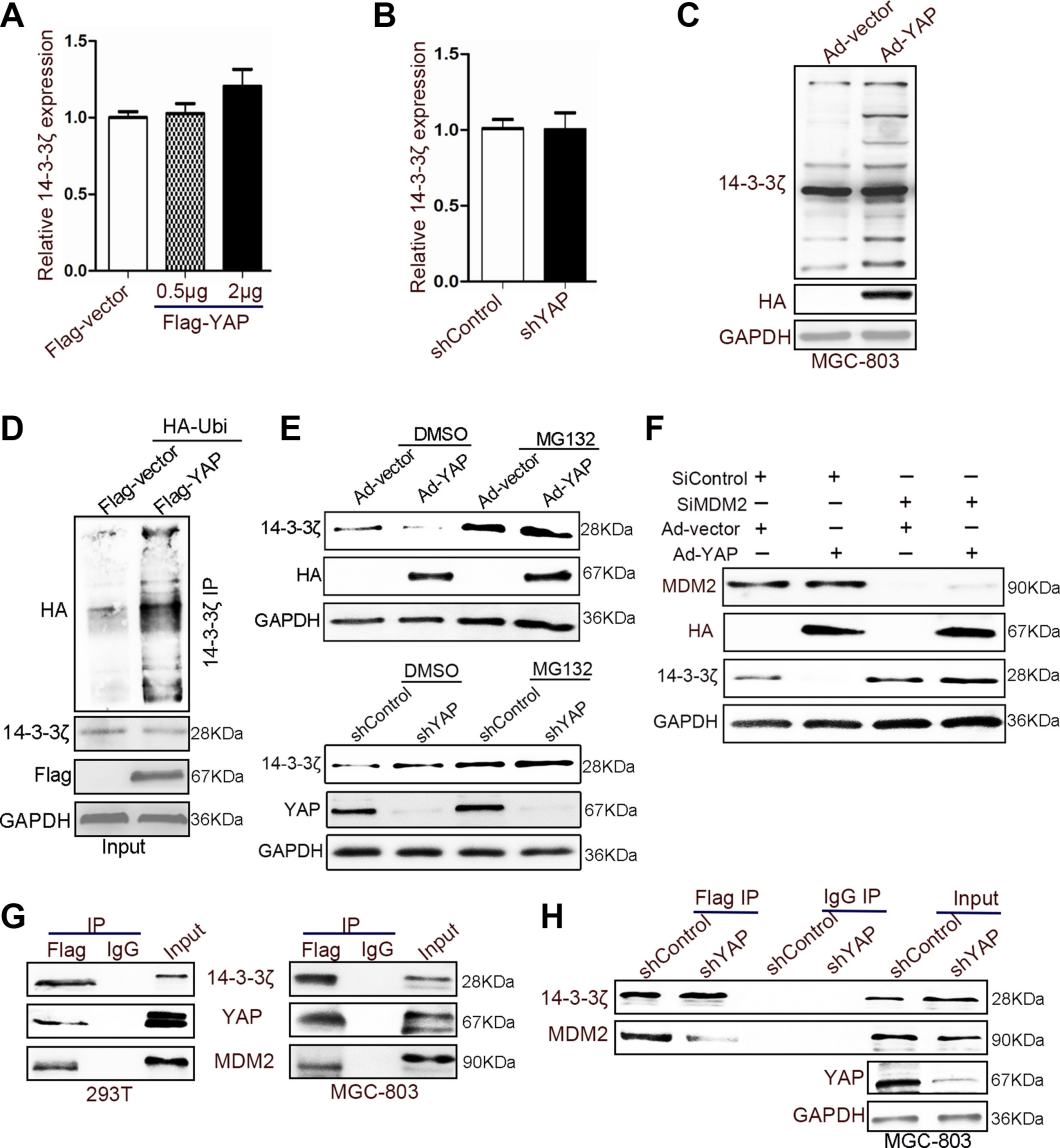
YAP recruited MDM2 to reduce the expression of 14-3-3ζ (**A**) Quantitative analyses of 14-3-3ζ mRNA in 293T cells transfected with Flag-vector or -YAP plasmid for 60 h. (**B**) The expression of 14-3-3ζ mRNA was detected in YAP-disrupted or control 293T cells through qRT-PCR after 48 h. (**C**) MGC-803 cells were transfected with vector and YAP-overexpressing adenovirus for 60 h. The cells were treated with MG132 (20 μM) for 2 h before harvesting, and the cell lysates were subjected to IB with 14-3-3ζ antibodies. (**D**) 293T cells were transfected with the indicated plasmids for 48 h. The cells were treated with MG132 (20 μM) for 2 h before harvesting, and the cell lysates were subjected to IP with 14-3-3ζ antibodies. Western blotting was used to detect 14-3-3ζ, and Flag (the tag of YAP overexpression). (**E**) Western blotting assay for 14-3-3ζ expression in YAP-disturbed or YAP-overexpressing adenovirus-transfected MGC-803 cells after treating with or without MG132 (20 μM). (**F**) The expression of HA (the tag of YAP overexpression), MDM2, and 14-3-3ζ was determined in YAP-overexpressing or nonoverexpressing MGC-803 cells with or without the disruption of MDM2 expression. (**G**) 14-3-3ζ was immunoprecipitated from 14-3-3ζ-Flag-overexpressing 293T and MGC-803 cells; YAP, p-LATS1, and 14-3-3ζ expressions were determined through western blotting. (**H**) YAP-disrupted or control MGC-803 cells were transfected with 14-3-3ζ-Flag-overexpressing adenovirus and subjected to IP using Flag antibodies or control IgG, followed by IB with YAP, MDM2, and 14-3-3ζ antibodies.

To explore the mechanism underlying the YAP-induced suppression of 14-3-3ζ expression, we detected the mRNA level of 14-3-3ζ in 293T cells in which YAP was overexpressed or knocked down. YAP could not inhibit the expression of the 14-3-3ζ mRNA level (Figure 6A and 6B), indicating that posttranslational regulation underlies the regulation of 14-3-3ζ by YAP. Shen et al. reported that YAP directly induced miRNA-130a expression to inhibit VGLL4 expression [32]. Therefore, we first hypothesized that YAP inhibits the expression of 14-3-3ζ by expressing microRNA (miRNA). We identified 12 miRNAs that can bind to the 3′-untranslated regions of 14-3-3ζ by using the TargetScan website (Supplementary Figure 6A). Only eight miRNAs were expressed in 293T cells; however, the overexpression and knockdown of YAP did not directly induce their expression in 293T cells (Supplementary Figure 6B and 6C). The inhibition of 14-3-3ζ by YAP was independent of its phosphorylation (Supplementary Figure 6D). This result suggested that YAP reduced 14-3-3ζ expression through a separate posttranslational regulation.

Proteins from the 14-3-3 family are a target of ATL31 ubiquitin ligase [33]. They can be suppressed by P53 through proteasome-mediated protein degradation [34]. Studies have shown that 14-3-3 proteins may be degraded through ubiquitination [33, 34]. Our results also indicated that YAP transfection increases 14-3-3ζ ubiquitination in MGC-803 and BGC-823 cells (Figure 6C and 6D). We confirmed this phenomenon by using the proteasome inhibitor MG132, which reversed the effect on the 14-3-3ζ induced by YAP overexpression or knockdown (Figure 6E). Furthermore, 14-3-3ζ was identified as an interacting protein for E3 ubiquitin ligase MDM2 [35]. The knockdown of MDM2 abolished the effect of YAP on 14-3-3ζ (Figure 6F). A previous study reported that YAP recruits an E3 ubiquitination enzyme (β-TrCP) to induce the degradation of β-catenin [36]. Furthermore, coimmunoprecipitation results revealed YAP, 14-3-3ζ, and MDM2 colocalization in 293T and MGC-803 cells (Figure 6G and Supplementary Figure 6E). Therefore, YAP may recruit MDM2 to 14-3-3ζ and promote 14-3-3ζ ubiquitination. The binding between 14-3-3ζ and MDM2 decreased when YAP expression was altered (Figure 6H).

### Verification of a YAP–14-3-3ζ negative feedback loop through murine models

**Figure 7:**
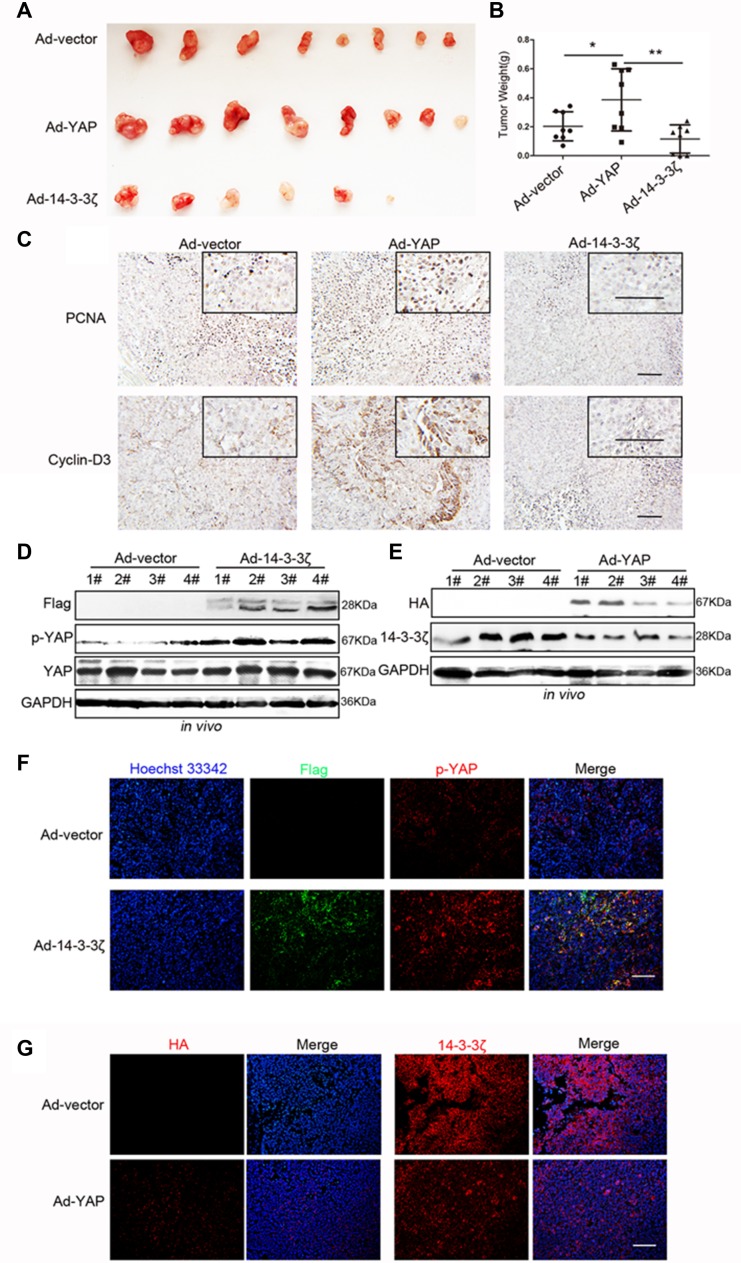
Verification of the YAP/14-3-3ζ regulatory relationship in a murine model (**A**) Representative images of subcutaneous tumors. (**B**) Tumor weight was evaluated in mice transfected with blank vector, YAP-overexpressing, and 14-3-3ζ-overexpressing MGC-803 cells. (**C**) Representative images of PCNA and Cyclin-D3 histochemical staining in the subcutaneous tumors tissues displayed in Figure 7A. Scale bar = 100 μm. (**D**) Western blotting assay for Flag (the tag for 14-3-3ζ overexpression expressed by the adenovirus vector), p-YAP, YAP, and GAPDH expressions in subcutaneous tumor tissue that was produced through injection with Ad-vector and Ad-14-3-3ζ transfected MGC-803 cells. (**E**) Western blotting assay for HA (the tag for YAP overexpression expressed by the adenovirus vector), p-YAP, YAP, and GAPDH expression in tumor tissue that was produced through subcutaneous injection with Ad-vector and Ad-YAP transfected MGC-803 cells. (**F**) Representative images of p-YAP (red) and Flag (green, representing 14-3-3ζ overexpression) immunofluorescence staining in mice tumor tissue overexpressing 14-3-3ζ or not overexpressing 14-3-3ζ. Scale bar = 100 μm. (**G**) Representative images of HA (left, representing YAP overexpression) and 14-3-3ζ (right) immunofluorescence staining in mice tumor tissue overexpressing YAP or not overexpressing YAP. Scale bar = 100 μm.

To demonstrate the regulatory relationship between YAP and 14-3-3ζ in murine models, we conducted *in vivo* tumorigenicity experiments through subcutaneous injections of the vectors, namely 14-3-3ζ- and YAP-overexpressing MGC-803 cells. As indicated in Figures 2 and 3, the overexpression of YAP *in vivo* promoted tumorigenesis whereas 14-3-3ζ overexpression *in vivo* inhibited tumorigenesis (Supplementary Figure 7, Figure 7A, and 7B). YAP promoted the expression of PCNA and Cyclin-D3, whereas 14-3-3ζ inhibited their expression (Figure 7C) Western blot and immunofluorescence assays revealed that 14-3-3ζ overexpression increased the phosphorylation of YAP at the Ser127 site in subcutaneous tumor tissue (Figure 7D and 7F). Moreover, the overexpression of YAP in subcutaneous tumor tissue reduced the expression of 14-3-3ζ (Figure 7E and 7G). These results confirmed the existence of a YAP–14-3-3ζ negative feedback loop in a murine model.

## DISCUSSION

The Hippo pathway plays a crucial role in organ size control and tissue homeostasis [37]. Studies of mouse models and clinical samples have confirmed the significance of this pathway in the development of human cancers [11, 38, 39]. Further investigation of the functions of this pathway and the regulatory mechanisms underlying it are required to facilitate the understanding of organ size control and identify new targets for cancer treatment [40]. YAP is considered to function as an oncoprotein in numerous tumor types [13–16]. The results of the present and a previous study have revealed that YAP is upregulated in gastric adenocarcinomas and promotes cell proliferation [17]. Zhang et al. first reported that VGLL4 functions as a novel inhibitor of the YAP–TEAD transcriptional complex in lung cancer [41]. They reported that GC tumor growth can be suppressed *in vitro* and *in vivo* by the peptide mimicking function of VGLL4 [42]. In addition, RUNX3 inhibits the TEAD–YAP oncogenic complex to inhibit GC growth [43]. These studies strongly indicate that the identification of a novel inhibitory molecule may provide a breakthrough in GC treatment. In this study, we first discovered the contrasting pattern of YAP and 14-3-3ζ expression and revealed a negative correlation between them in GC tissues. Therefore, we suspected that 14-3-3ζ may be another inhibitory molecule of YAP in GC. Further research revealed that 14-3-3ζ inhibited the activation of YAP by inducing its phosphorylation in GC cells.

Proteins in the 14-3-3 family can bind to hundreds of partners [22]. Previous studies have reported that the role of 14-3-3 proteins in the Hippo pathway was only to bind to p127-YAP and induce the cytoplasmic retention of YAP [10, 12, 26, 27]. However, our study revealed that, in addition to inducing the YAP location, the overexpression of 14-3-3ζ promoted YAP phosphorylation. Furthermore, 14-3-3 proteins regulated target proteins in several ways, such as by changing the protein conformation, affecting protein activity or stability, altering protein subcellular localization, and facilitating protein complex formation [20, 21]. Our previous study demonstrated that 14-3-3ζ induced YAP phosphorylation by mediating the binding of YAP and p-LATS in the Hippo pathway. In brief, the binding of p-LATS and YAP is dependent on 14-3-3ζ and is required for YAP phosphorylation in 293T cells [29]. This finding strongly suggests that 14-3-3ζ is not just a companion molecule but also a regulator of the Hippo pathway. Our studies have clarified the novel role of 14-3-3ζ in the Hippo pathway.

The exact role of 14-3-3 proteins in cancer progression is unclear. For example, 14-3-3σ was initially considered a tumor suppressor in breast and prostate cancers [44–46]. However, 14-3-3σ is also associated with relatively unfavorable prognoses in hepatocellular carcinoma, indicating that 14-3-3σ is an enhancer of liver cancer [47]. Thus, 14-3-3σ is a “double-edged sword” in human cancers [48]. In addition, 14-3-3ζ is considered to be a central cellular hub protein that regulates the multiple signaling pathways involved in cancer progression [49]. In prostate cancer, 14-3-3ζ is overexpressed and facilitates cancer progression [22]. Moreover, 14-3-3ζ promotes the phosphorylation of AKT by binding to the p85 regulatory subunit, which primes human breast cancer cells for invasion in response to ionizing radiation [23, 24]. A recent study revealed that 14-3-3ζ alters the function of TGF-β from that of a tumor suppressor to a metastasis promoter by changing the Smad partner from p53 to Gli2 [25]. The aforementioned studies clearly demonstrate that 14-3-3ζ is a cancer promoter. However, approximately 35.7% of GC tissue samples expressed lower 14-3-3ζ than did their paired adjacent tissues, and 14-3-3ζ overexpression significantly inhibited cell proliferation in multiple GC cell lines; the aforementioned characterization of 14-3-3ζ cannot explain these results, which suggested that 14-3-3ζ may be also a double-edged sword for GC.

Because of the vital role of 14-3-3 proteins in the Hippo signaling pathway, understanding the action of 14-3-3ζ on YAP is crucial. However, few studies have focused on clarifying this action. Our review of the relevant literature indicated that this study is the first to report that YAP reduced the expression of 14-3-3ζ but not its mRNA level. On the basis that YAP can directly promote miRNA-130a transcription and inhibit VGLL4 expression [32], we hypothesized that YAP inhibited 14-3-3ζ gene translation by first expressing some miRNAs. However, YAP did not induce the expression of miRNAs that can repress the translation of the 14-3-3ζ protein. Furthermore, 14-3-3 proteins can be degraded through ubiquitination [33, 34], and 14-3-3ζ was an interacting protein for the E3 ubiquitin ligase MDM2 [35]. MDM2 inhibits the P53 pathway by inducing P53 ubiquitination [50]. However, whether 14-3-3ζ can be ubiquitinated by MDM2 remains unknown. Our results reveal that the knockdown of MDM2 abolished the reduction of 14-3-3ζ induced by YAP in MGC-803 cells. We observed the novel finding that MDM2 is involved in the Hippo pathway through the regulation of the YAP–14-3-3ζ axis.

In summary, we first revealed that 14-3-3ζ and YAP formed a special negative feedback loop in GC and affected cell proliferation. The 14-3-3ζ protein induced the cytoplasmic retention of YAP and inhibited its transcriptional activity by mediating the binding of YAP and p-LATS. By contrast, YAP recruited MDM2 to 14-3-3ζ and reduced the stability of 14-3-3ζ.

## MATERIALS AND METHODS

### Human gastric cancer samples

The primary GC tissues and their matching adjacent noncancerous tissues (located more than 5–10 cm away from the primary site) were collected from patients with GC undergoing surgery at the Affiliated Hospital of Jiangsu University in Zhenjiang, Jiangsu, China.

Tissue microarrays of 178 primary gastric tumor cases were used for detecting YAP and 14-3-3ζ expression; the samples were preserved in the Gastric Cancer Tissue Bank at the Department of Oncology, Changzheng Hospital (Shanghai, China). All the cases underwent curative resection. In addition, all tissue specimens for this study were obtained with patients’ informed consent, and the study protocols were approved by the institutional review boards of Chang Zheng Hospital and Jiangsu University.

### Cell lines and cell culture

The human GC cell lines SGC-7901, AGS, HGC-27, BGC-823, MKN-45, and MGC80-3 were purchased from the Institute of Biochemistry and Cell Biology at the Chinese Academy of Sciences (Shanghai, China). SGC-7901, AGS, and HGC-27 cells were cultured in RPMI-1640 medium (Gibco, Grand Island, NY, USA). BGC-823, MKN-45, and MGC80-3 cells were propagated in a high-glucose Dulbecco's modified Eagle's medium (Gibco, Grand Island, NY, USA). All media were supplemented with 10% fetal bovine serum. The cells were cultured at 37°C in humidified air with 5% CO_2_.

### Immunohistochemistry

Immunohistochemistry was used to detect YAP (1:50; Bioworld, Louis Park, MN, USA) and 14-3-3ζ (1:100; Bioworld, Louis Park, MN, USA). Images were sequentially acquired through microscopy (Nikon, Tokyo, Japan).

### Colony formation assay

The cells were harvested and seeded into a 6-well plate (1000 cells/well) and incubated at 37°C in a 5% CO_2_ humidified incubator for the indicated time. At the end of the incubation period, the cultures were fixed with 4% paraformaldehyde and stained with crystal violet.

### Western blotting

Cell and tissue lysates were extracted in a lysis buffer containing 50 mM sodium chloride (NaCl), 1 mM ethylene glycol tetraacetic acid, 0.1% sodium dodecyl sulfate (SDS), 1 mM sodium fluoride, 1 mM sodium orthovanadate, 1 mg/mL aprotinin, and 1 mg/mL leupeptin in 10 mM Tris buffer (pH 7.4) and proteinase inhibitor (1 mM n-phenylmethanesulfonyl fluoride). Equal amounts of the total protein were separated on a 12% SDS-polyacrylamide gel and transferred to nitrocellulose membranes (Millipore). The membranes were incubated overnight with monoclonal antibodies against GAPDH, PCNA, cyclin-D1, cyclin-D3, YAP, p-YAP, 14-3-3ζ, LATS1, MDM2, histone, Flag, and haemagglutinin (HA) (Supplementary Table 1). The membrane was washed three times with tris-buffered saline and Tween and incubated with secondary antibodies (Bioworld, Louis Park, MN, USA) at 37°C for 1 h. The signals were visualized using a Luminata crescendo western horseradish peroxidase substrate (Millipore, Billerica, MA, USA).

### RNA extraction, RT-PCR, and real-time RT-PCR (mRNA)

Total RNA was isolated from skin cells and tissues by using Trizol reagents (Invitrogen, Carlsbad, CA, USA). Two-microgram aliquots of RNA were synthesized according to the manufacturer's protocol (Vazyme, Nanjing, China). Real-time polymerase chain reaction (PCR) reactions were conducted using the QuantiTect SYBR Green PCR kit (Toyobo). The primer sequences are listed in Supplementary Table 2.

### RT-PCR and real-time RT-PCR for miRNAs

The poly(A) tailing kit (New England Biolabs, MA, USA) was used for detecting miRNA as follows: 1 μg of total RNA was mixed with 2 μL poly(A) buffer (10 nM), 2 μL ATP (10 nM), and 0.5 μL *Escherichia coli* poly(A) polymerase I. The total volume was adjusted to 20 μL with RNase-free ddH_2_O. Furthermore, single-stranded cDNA was obtained from RNA by using reverse transcriptase (Takara, China). A total of 0.5 μg of total poly(A)-tailing RNA was mixed with 2 μL of M-MLV buffer (5×), 0.5 μL of a dNTP mixture (10 mM), 0.25 μL of an RNase inhibitor (40 U/μL), 0.25 μL of RTase M-MLV (RNase H; 200 U/μL), and 1 μL of adaptor(dT) 15 (50 μL M). Furthermore, the total volume was adjusted to 20 μL with RNase-free ddH_2_O. Reverse transcription was performed at 42°C for 60 min, followed by an inactivation reaction at 70°C for 15 min. The PCR mixture contained 10 μL of the qPCR master mix (2×; Bio-Rad, CA, USA) and 1 μL of cDNA; the total volume was increased to 20 μL with ddH_2_O. The primer powder was fixed to the bottom of a 96-well plate and 20 μL of the PCR mixture was added to each well. Reverse transcription (RT)-PCR was performed using the CFX96 real-time instrument (Bio-Rad). Furthermore, real-time RT-PCR was performed using the SYBR Green q-PCR Super mix (Bio-Rad) with specific primers (Supplementary Table 2). The thermal cycle parameters were as follows: 95°C for 5 min, 40 cycles at 95°C for 15 s, 60°C for 15 s, 72°C for 20 s, and a 65–95°C drawing dissociation curve. The expression of each gene was defined from the threshold cycle (Ct), and the melting temperatures were recorded. The relative changes in miRNA expression were analyzed through the ΔΔCt method.

### Cytoplasm and nuclear fractionation

Cytoplasm and nuclear fractionation were performed according to the manufacturer's instructions (Vazyme, Nanjing, China). In brief, the cells were harvested and washed once with cold phosphate-buffered saline (PBS). The cells were then suspended in isolation buffer A mixed with protease inhibitors and rotated at 4°C for 1 min. After 12,000 × g centrifugation at 4°C for 5 min, the supernatant, containing the cytoplasm fraction, was collected. The remaining cell debris were suspended in isolation buffer B mixed with protease inhibitors and centrifuged three times every 10 min at 4°C for 1 min. The samples for cytoplasm and nuclear fractionation were stored at −80°C in preparation for western blot detection.

### Luciferase assay

For the YAP reporter assay, HEK293T cells were seeded in 24-well plates. A combination of 5 × UAS-luciferase reporter, b-actin-Renilla, 3 × Flag-YAP, GAL4-TEAD4, and other indicated plasmids was cotransfected as indicated in Figure 4O. After 48 h following transfection, cells were lysed and luciferase activity was assayed using an enhanced luciferase assay kit obtained from Promega following the manufacturer's instructions. All luciferase activities were normalized to Renilla activity.

### Cell counting assay

In total, 5 × 10^3^ MGC-803, HGC-27, and MKN-45 cells were plated in each well of the 24-well plates for cell counting assay. The number of cells in each well was counted in triplicate at different time points.

### Lentiviral knockdown of target genes in cells

The lentiviral expression vector containing the shRNA sequence (Sigma) was selected for target-specific gene silencing; the target genes are listed in Supplementary Table 3. Lenti-GFP–shRNA was used as the negative control vector. The shRNA sequences of control and target-specific genes are listed in Supplementary Table 3. The shRNA lentiviral vectors were generated by ligating the vector Tet-pLKO-puro; these lentiviral vectors were produced using a lentivirus packaging mix (ViraPower, Invitrogen). In addition, stable cell lines were obtained after selection with 1 μg/mL of puromycin (Invitrogen) for 15 days. The expression of shRNA was induced by adding 80 μg/mL doxycycline for 2 days. The efficiency of wnt4 knockdown was evaluated through real-time quantitative RT-PCR and western blotting.

### Immunoprecipitation

The cells were lysed in a co-immunoprecipitation (IP) buffer (10 mM HEPES [pH 8.0], 300 mM NaCl, 0.1 mM EDTA, 20% glycerol, 0.2% NP-40, and protease and phosphatase inhibitors). The lysates were centrifuged and cleared through incubation with 25 μL of a protein A/G gel for 1.5 h at 4°C. The precleared supernatant was then subjected to IP using the indicated antibodies at 4°C overnight. The protein complexes were then collected by incubating with 30 μL of protein A/G gel for 2 h at 4°C. The collected protein complexes were washed six times with a co-IP buffer and analyzed through western blotting.

### siRNA transfection

Chemically synthesized MDM2 siRNAs and the matching scramble control siRNAs were purchased from GenePharma Co., Ltd (Shanghai, China; Supplementary Table 3). The siRNAs were transiently transfected into SGC-7901 and MGC803 cells by using Lipofectamine 2000 reagent (Invitrogen, Carlsbad, CA, USA) according to the manufacturer's instructions. The cells were plated in 6-well plates at a density of 1 × 10^5^ cells/well. The transfection reagent and scrambled siRNA-transfected cells were used as controls.

### *In vivo* tumorigenicity

Twelve male BALB/c nu/nu mice (Laboratory Animal Center of Shanghai, Academy of Science, Shanghai, China) aged 4–6 weeks were randomly divided into three groups (four mice per group). All the groups received subcutaneous injections of the vector, namely 14-3-3ζ- and YAP-overexpressing MGC-803 cells (2 × 10^6^ cells in 200 μL PBS) on both sides of their upper limbs. Tumor growth was evaluated through weight measurement.

### Cell scratch assay

MGC-803 cells were seeded at a density of 2 × 10^5^ cells/well in 6-well plates and incubated at 37°C in 5% CO_2_ for 24 h to create confluent monolayers. The monolayers were scratched with a sterile pipette tip. To measure cell mobility, we stained the cells with crystal violet and obtained images in five random fields at 24 h after scratching.

### Statistical analysis

All data are presented as means ± standard deviation (SD). The correlation of the expression levels of YAP and 14-3-3ζ in gastric tissue microarrays was analyzed using the chi-square test (SPSS Statistics version 20 software). Overall survival curves were plotted according to the Kaplan–Meier method, with the log-rank test was applied for comparison. The statistically significant differences between groups were assessed using analysis of variance and *t*-tests with the Prism software package (GraphPad, San Diego, USA). A *p* value of less than 0.05 was considered statistically significant.

## SUPPLEMENTARY MATERIALS FIGURES AND TABLES


