# Supplementary material for: Identification of a novel YAP–14-3-3ζ negative feedback loop in gastric cancer
Source: Oncotarget. 2017 May 19;8(42):71894–910. doi: 10.18632/oncotarget.18011 (PMC5641098; doi:10.18632/oncotarget.18011)
Supplement: Supplementary file 1 [file oncotarget-08-71894-s001.pdf]

Identification of a novel YAP–14-3-3ζ negative feedback loop in gastric cancer

SUPPLEMENTARY MATERIALS

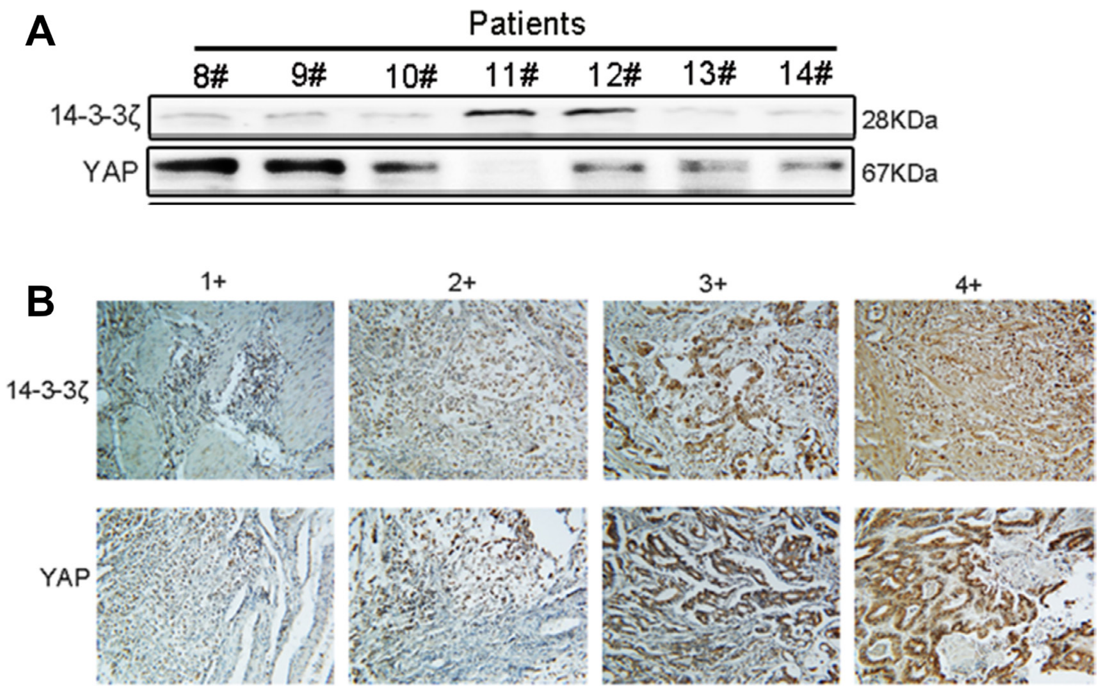

Supplementary Figure 1: Western blotting assay of YAP and 14-3-3ζ protein levels in gastric cancer. (**A**) The grading standard of YAP and 14-3-3ζ expression (**B**).

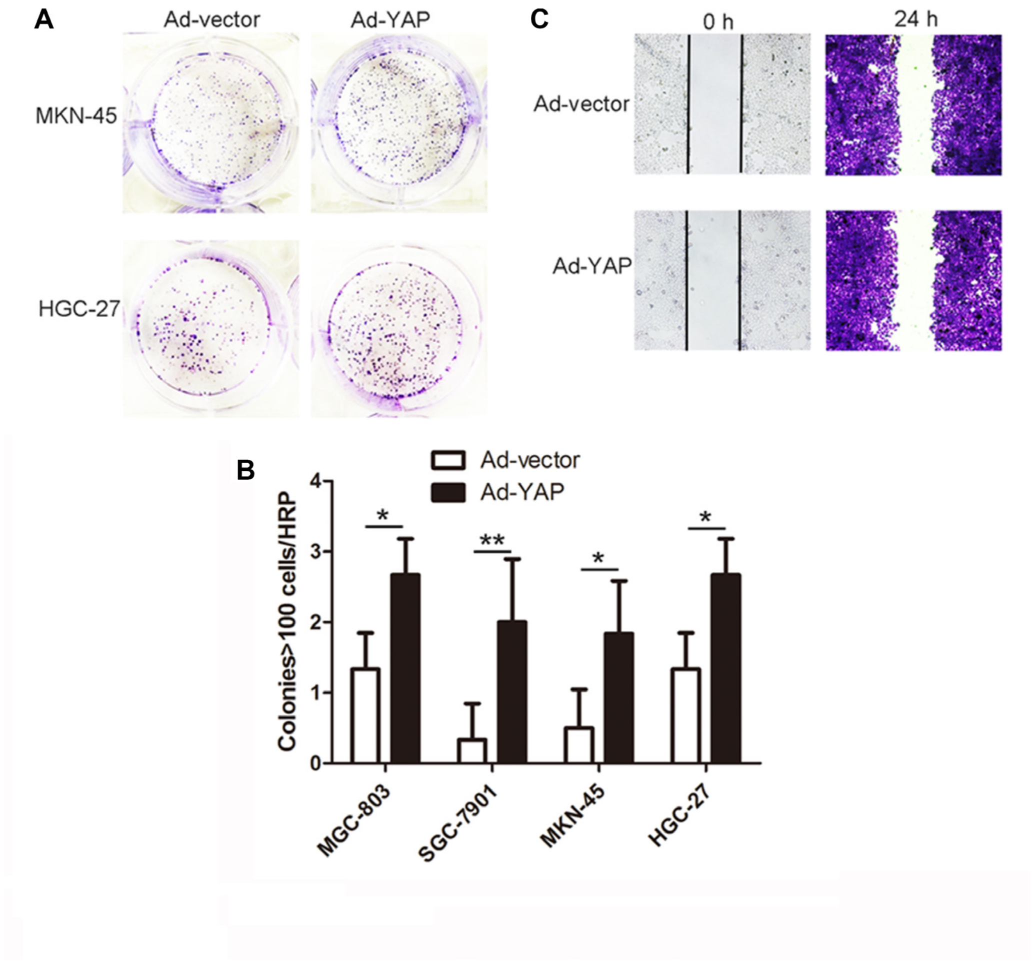

Supplementary Figure 2: Representative images of colony formation in vector and YAP-overexpressing adenovirus transfected MKN-45 and HGC-27 cells. The transfected cells were cultured for 5 days. (**A**) Statistical analysis of colony formation assay in Figure 2C and Supplementary Figure 2A ($n = 6$; $*p < 0.05$; $**p < 0.01$) (**B**) The migration ability of MGC-803 transfected with vector and YAP-overexpressing adenovirus was determined by scratch assay (**C**).

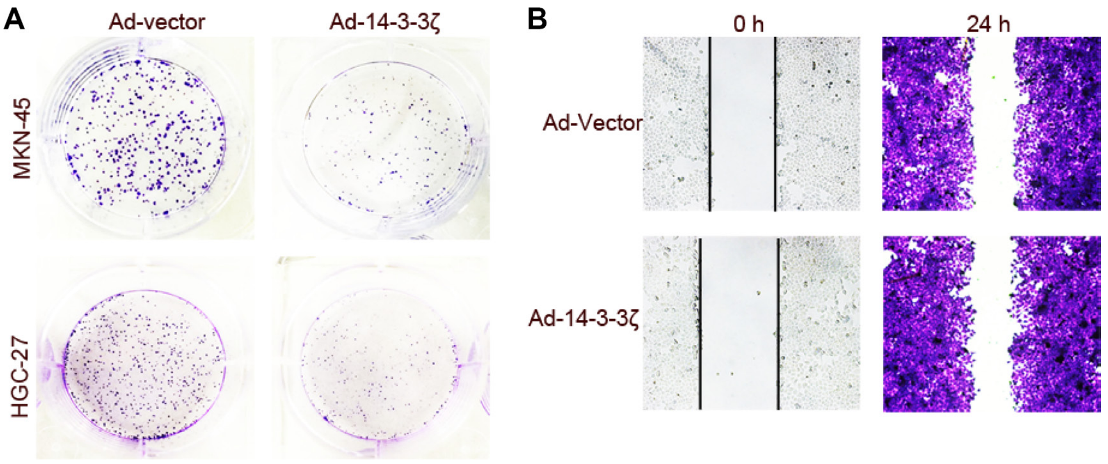

Supplementary Figure 3: Representative images of colony formation in vector and 14-3-3ζ-overexpressing adenovirus transfected MKN-45 and HGC-27 cells. The transfected cells were cultured for 10 days. (**A**) the migration ability of MGC-803 transfected with vector and 14-3-3ζ-overexpressing adenovirus was determined by scratch assay (**B**).

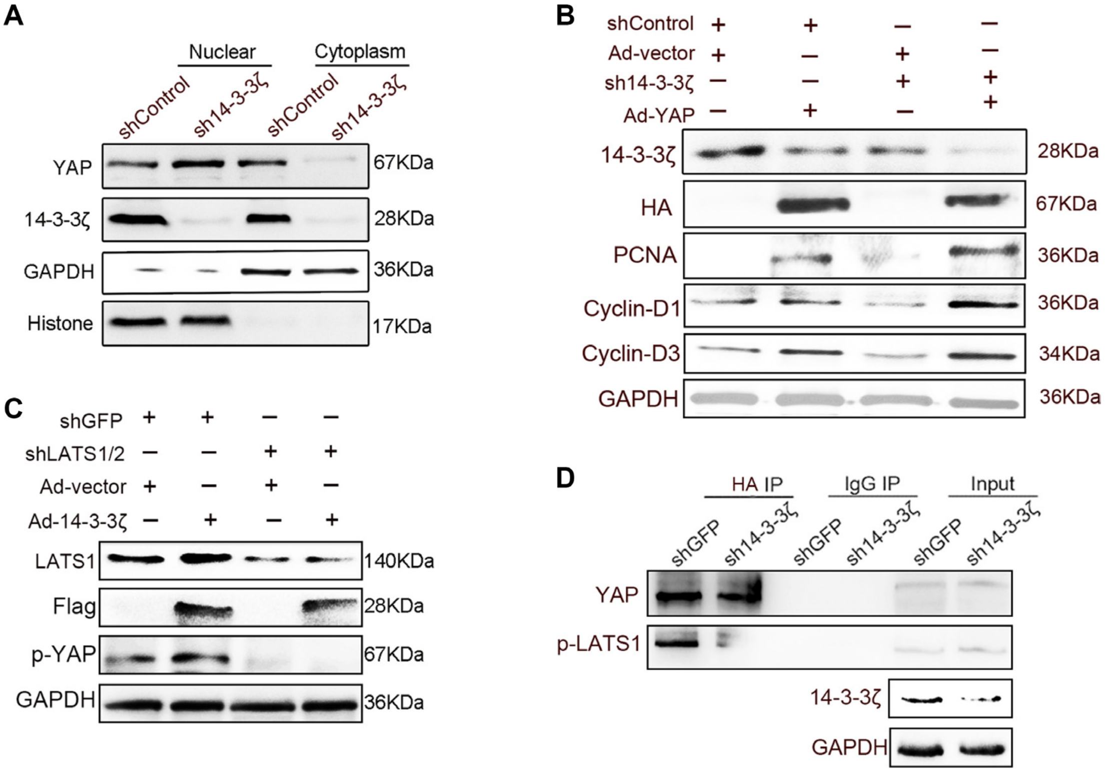

Supplementary Figure 4: Cytoplasmic and nuclear fractions were prepared from MGC-803 cells transfected with shControl or sh14-3-3ζ lentiviral and YAP protein levels were determined by western blotting (**A**) western blot assay for proliferation index (Cyclin-D3, Cyclin-D1, PCNA), HA and 14-3-3ζ in MGC-803 cells treated with blank-vector or YAP over-expressing adenovirus and shControl or sh14-3-3ζ lentiviral. (**B**) Expression of LATS1, Flag, p-YAP1, and YAP1 was determined in 14-3-3ζ-overexpressing or not overexpressing MGC-803 cells with or without the disruption of LATS expression (**C**) 14-3-3 fdisrupted and control MGC-803 cells were transfected with Flag-YAP plasmid and subjected to immunoprecipitation(IP) using Flag antibody or control IgG, followed by immunoblotting(IB) with YAP, p-LATS1 or 14-3-3 f antibodies (**D**).

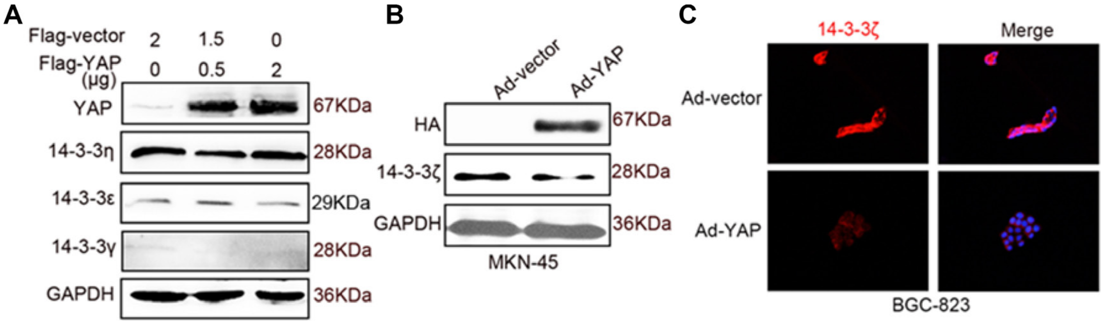

Supplementary Figure 5: Western blotting assay for 14-3-3η, 14-3-3ε, 14-3-3γ and 14-3-3σ expression after transfecting with Flag-vector or Flag-YAP plasmid in 293T cells at 60 h. (**A**) Western blotting assay for 14-3-3ζ expression in MKN-45 cells after transfecting with vector or YAP overexpressing adenovirus. (**B**) Representative images of YAP immunofluorescence treating with Ad-vector or Ad- YAP (**C**).

A

	predicted consequential pairing of target region (top) and miRNA (bottom)	seed match	site-type contri-bution	3' pairing contri-bution	local AU contri-bution	position contri-bution	TA contribution	SPS contribution	context+ score	context+ score percentile	conserved branch length	P_{CT}
Position 613-619 of YWHAZ 3' UTR<br>hsa-miR-761	5' ...UUAUUUGUAAAGUUA---CCUGCUGU...<br>3' ACACAGUCAAAGUGGGACGACG	7mer-m8	-0.120	-0.025	-0.105	0.025	0.031	-0.064	-0.26	93	2.569	0.48
Position 613-619 of YWHAZ 3' UTR<br>hsa-miR-214	5' ...UUAUUUGUAAAGUUACCUGCUGU...<br>3' UGACGGACAGACAC--GGACGACA	7mer-m8	-0.120	-0.016	-0.105	0.025	0.031	-0.064	-0.25	92	2.569	0.48
Position 613-619 of YWHAZ 3' UTR<br>hsa-miR-3619-5p	5' ...UUAUUUGUAAAGUUACCUGCUGU...<br>3' CGACGUGGUCGGACGGACGACU	7mer-m8	-0.120	0.003	-0.105	0.025	0.031	-0.064	-0.23	90	2.569	0.48
Position 1100-1107 of YWHAZ 3' UTR<br>hsa-miR-22	5' ...UCCUUAUUCCCUCUUGGCAGCUA...<br>3' UGUCAAGAAGUUGA--CCGUCGAA	8mer	-0.247	-0.018	-0.038	0.141	0.003	-0.143	-0.30	92	1.387	0.46
Position 590-597 of YWHAZ 3' UTR<br>hsa-miR-613	5' ...GGGCAGAAACGGUUCACAUUCCA...<br>3' CCGUUUCUUCCUUGUAAGGA	8mer	-0.247	-0.008	-0.007	0.035	0.010	0.016	-0.20	88	2.569	0.87
Position 590-597 of YWHAZ 3' UTR<br>hsa-miR-1	5' ...GGGCAGAAACGGUUCACAUUCCA...<br>3' UAUGUAUGAAGAAAUGUAAGGU	8mer	-0.247	0.003	-0.007	0.035	0.010	0.016	-0.19	86	2.569	0.87
Position 590-597 of YWHAZ 3' UTR<br>hsa-miR-206	5' ...GGGCAGAAACGGUUCACAUUCCA...<br>3' GGUGUGUGAAGGAAUGUAAGGU	8mer	-0.247	0.003	-0.007	0.035	0.010	0.016	-0.19	86	2.569	0.87
Position 85-91 of YWHAZ 3' UTR<br>hsa-miR-30d	5' ...CCACAAAUAGUUUUUUGUUUACG...<br>3' GAAGGUCAGCCCCUACAAAUGU	7mer-m8	-0.120	-0.007	-0.087	-0.050	0.009	0.062	-0.19	88	2.395	0.71
Position 85-91 of YWHAZ 3' UTR<br>hsa-miR-30a	5' ...CCACAAAUAGUUUUUUGUUUACG...<br>3' GAAGGUCAGCUCCUACAAAUGU	7mer-m8	-0.120	-0.007	-0.087	-0.050	0.009	0.062	-0.19	88	2.395	0.71
Position 85-91 of YWHAZ 3' UTR<br>hsa-miR-30b	5' ...CCACAAAUAGUUUUUUGUUUACG...<br>3' UCGACUCACAUCCUACAAAUGU	7mer-m8	-0.120	-0.007	-0.087	-0.050	0.009	0.062	-0.19	87	2.395	0.71
Position 85-91 of YWHAZ 3' UTR<br>hsa-miR-30c	5' ...CCACAAAUAGUUUUUUGUUUACG...<br>3' CGACUCUCACAUCCUACAAAUGU	7mer-m8	-0.120	-0.007	-0.087	-0.050	0.009	0.062	-0.19	87	2.395	0.71
Position 85-91 of YWHAZ 3' UTR<br>hsa-miR-30e	5' ...CCACAAAUAGUUUUUUGUUUACG...<br>3' GAAGGUCAGUUCCUACAAAUGU	7mer-m8	-0.120	-0.007	-0.087	-0.050	0.009	0.062	-0.19	87	2.395	0.71

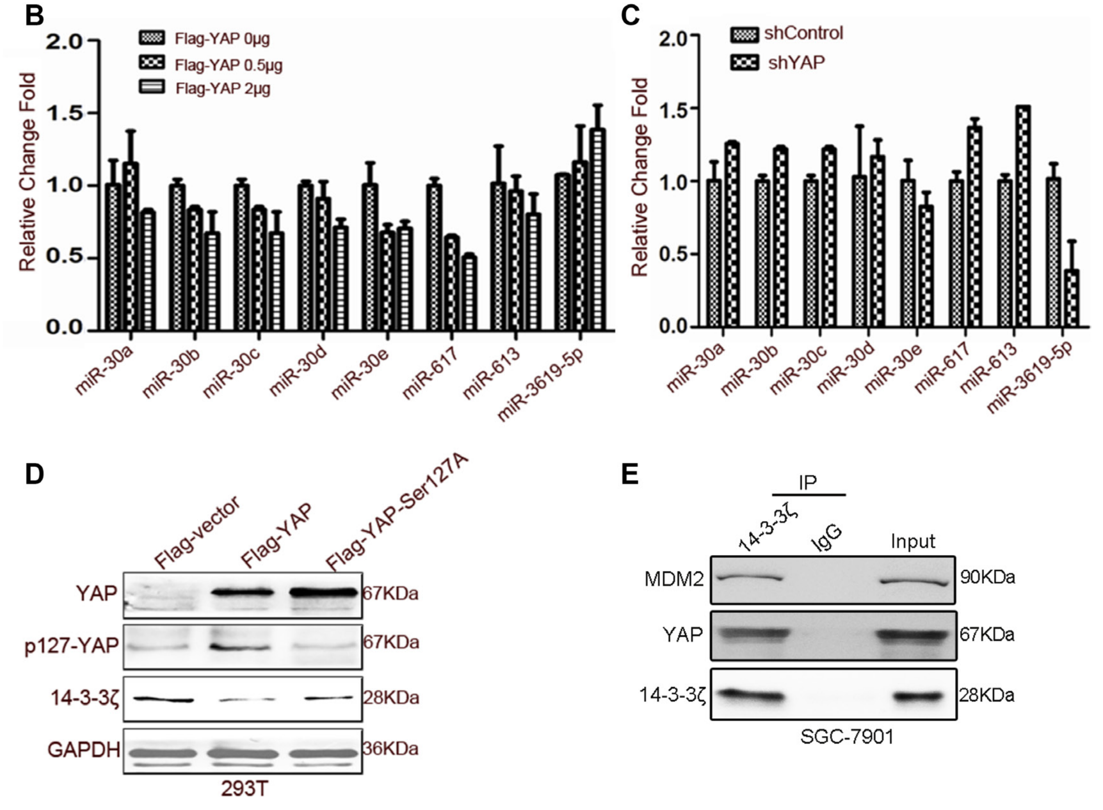

Supplementary Figure 6: Quantitative analyses of miR-30a, miR-30b, miR-30c, miR-30d, miR-30e, miR-617, miR-613 and miR-3619-5p in 293T cells transfected with Flag-vector or Flag-YAP plasmid at 60 h. (**A**) Quantitative analyses of miR-30a, miR-30b, miR-30c, miR-30d, miR-30e, miR-617, miR-613 and miR-3619-5p in YAP-overexpressed or control 293T cells. (**B**) Quantitative analyses of miR-30a, miR-30b, miR-30c, miR-30d, miR-30e, miR-617, miR-613 and miR-3619-5p in YAP-disrupted or control 293T cells. (**C**) Western blotting assay for 14-3-3ζ, p-YAP and YAP expression in 293T transfected with Flag-vector or Flag-YAP or Flag-YAP-Ser127A (Serine 127 site was mutated to Alanine) (**D**) Endogenous interaction between YAP, MDM2 and 14-3-3ζwas detected in MGC-803 cells (**E**).

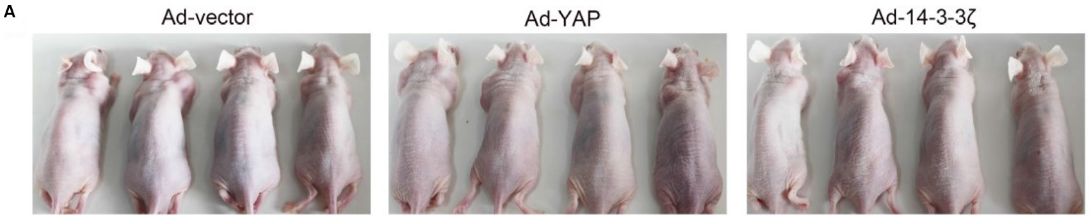

Supplementary Figure 7: Representative images of tumor-bearing mice (**A**).

Supplementary Table 1: Antibodies

Antibody	Catalog number	Detection	Manufacturer
GAPDH	CW0100A	WB	KangCheng
MDM2	BS1447	WB	Bioworld
Cyclin D1	P24385	WB	Bioworld
PCNA	BS1289	WB	Bioworld
Cyclin-D3	P30281	WB/IHC	Bioworld
14-3-3ζ	BS1001	WB/IP/ IHC	Bioworld
14-3-3 γ	BS2512	WB	Bioworld
14-3-3 ε	BS6109	WB	Bioworld
14-3-3 η	BS2384	WB	Bioworld
YAP	BS2000	WB/IHC/IF	Bioworld
p-YAP	4911	WB/IHC/IF	Cell signaling technology
LATS1	3477	WB	Cell signaling technology
Histone	sc-8655	WB	Santa Cruz Biotechnology
Flag	F1804	WB/IP/IF	Sigma
HA(Rabbit)	3724	WB/IP/IF	Cell signaling technology
HA(Mice)	H3663	WB	Sigma

Supplementary Table 2: Sequences of real-time PCR primers

mRNA/miRNA	Primer	Sequences (5′-3 ′)
Human-14-3-3ζ	Forward	CAGATGGCTCGAGAATACAG
	Reverse	CCTCAGCCAAGTAACGGTAG
Human-CTGF	Forward	ATCTTCGGTGGTACGGTGT
	Reverse	GTGTCTTCCAGTCGGTAAGC
Human-Cy61	Forward	GCTTGGCGCAGACCTTACAGCA
	Reverse	TTGACCAGGCTGGCGCTCTC
Human/ β-actin	Forward	GACCTGTACGCCAACACAGT
	Reverse	CTCAGGAGGAGCAATGATCT
hsa-miR-613	Forward	AGCAGGGTGAAACTGACACA
	Reverse	GCGAGCACAGAATTAATACGAC
hsa-miR-3619-5p	Forward	TCAGCAGGCAGGCTGGTG
	Reverse	GCGAGCACAGAATTAATACGAC
hsa-miR-214	Forward	ACAGCAGGCACAGACAGGC
	Reverse	GCGAGCACAGAATTAATACGAC
hsa-miR-1	Forward	TGGAATGTAAAGAAGTATGTAT
	Reverse	GCGAGCACAGAATTAATACGAC
hsa-miR-206	Forward	TGGAATGTAAGGAAGTGTGTGG
	Reverse	GCGAGCACAGAATTAATACGAC
hsa-miR-30d	Forward	TGTAAACATCCCCGACTGGAAG
	Reverse	GCGAGCACAGAATTAATACGAC
hsa-miR-30a	Forward	TGTAAACATCCTCGACTGGAAG
	Reverse	GCGAGCACAGAATTAATACGAC
hsa-miR-22	Forward	AAGCTGCCAGTTGAAGAACTGT
	Reverse	GCGAGCACAGAATTAATACGAC
miRNA-761	Forward	AGCAGGGTGAAACTGACACA
	Reverse	GCGAGCACAGAATTAATACGAC
miRNA-30b	Forward	TGTAAACATCCTACACTCAGCT
	Reverse	GCGAGCACAGAATTAATACGAC
miRNA-30c	Forward	TGTAAACATCCTACACTCTCAGC
	Reverse	GCGAGCACAGAATTAATACGAC
miRNA-30e	Forward	TGTAAACATCCTTGACTGGAAG
	Reverse	GCGAGCACAGAATTAATACGAC

Supplementary Table 3: ShRNA and siRNA Oligonucleotides

Target Gene	Sequences (5′-3′)
Human-sh14-3-3ζ	Forward CCGGGCAGAGAGCAAAGTCTTCTATCTCGAGATAGAAGACTTTGCTCTCTGCTTTTTG
	Reverse AATTCAAAAAGCAGAGAGCAAAGTCTTCTATCTCGAGATAGAAGACTTTGCTCTCTGC
Human-shYAP	Forward CCGGGCCACCAAGCTAGATAAAGAACTCGAGTTCTTTATCTAGCTTGGTGGCTTTTTG
	Reverse AATTCAAAAAGCCACCAAGCTAGATAAAGAACTCGAGTTCTTTATCTAGCTTGGTGGC
Human-shYAP-3′UTR	Forward CCGGCCCAGTTAAATGTTCACCAATCTCGAGATTGGTGAACATTTAACTGGGTTTTTG
	Reverse AATTCAAAAACCCAGTTAAATGTTCACCAATCTCGAGATTGGTGAACATTTAACTGGG
Human-siMDM2	Forward GCCAGUAUAUUAUGACUAATT
	Reverse UUAGUCAUAAUAUACUGGCTT
Human-siNC	Forward UUCUCCGAACGUGUCACGUTT
	Reverse ACGUGACACGUUCGGAGAATT

Supplementary Table 4: Protease inhibitor

Inhibitor	Target molecular	Manufacturer
MG132	Protease	Sigma

Supplementary Table 5: Adenovirus vector information

Name	Vector information
Ad-vector (empty control vector)	CMV-eGFP
Ad-YAP (YAP overexpression vector)	pAV(Exp)-CMV>YAP/HA-IRES-eGFP
Ad-14-3-3ζ (14-3-3ζ overexpression vector)	pAV(Exp)-CMV>14-3-3ζ/FLAG-IRES-eGFP
